# Pseudorabies gD protein protects mice and piglets against lethal doses of pseudorabies virus

**DOI:** 10.3389/fmicb.2023.1288458

**Published:** 2023-11-06

**Authors:** Mengpo Zhao, Jing Chen, Shengjun Luo, Renhe Yan, Pian Zhang, Zhaowen Ren, Xiaofan Chen, Gang Wang, Hua Xiang, Rujian Cai, Yuan Huang, Na Li, Hongwei Li, Zi-Guo Yuan, Xiaohu Wang

**Affiliations:** ^1^Key Laboratory of Livestock Disease Prevention of Guangdong Province, Scientific Observation and Key Laboratory for Prevention and Control of Avian Influenza and Other Major Poultry Diseases, Ministry of Agriculture and Rural Affairs, Institute of Animal Health, Guangdong Academy of Agricultural Sciences, Guangzhou, China; ^2^College of Veterinary Medicine, South China Agricultural University, Guangzhou, China; ^3^Department of Research and Development, Guangzhou Bioneeds Biotechnology Co., LTD, Guangzhou, China; ^4^School of Laboratory and Biotechnology, Southern Medical University, Guangzhou, China

**Keywords:** pseudorabies virus, 293T cells, recombinant gD protein, subunit vaccine, immune protection

## Abstract

**Introduction:**

Pseudorabies (PR) is a highly contagious viral disease caused by the pseudorabies virus (PRV), which can cause disease in a wide range of domestic and wild animals. Studies have shown that new mutant strains have emerged in pig farms in many regions and that commercial inactivated and live attenuated vaccines are becoming less effective at protecting pigs.

**Methods:**

Porcine pseudorabies glycoprotein D (gD) gene (GenBank: QEY95774.1) with hexa-His tag to the C terminus for further purification processes was cloned into the lentiviral expression plasmid pLV-CMV-eGFP by restriction enzyme, the resulting plasmid was designated as pLV-CMV-gD. HEK-293T cells with robust and stable expression of recombinant gD protein was established by infection with recombinant lentivirus vector pLV-CMV-gD. We expressed porcine pseudorabies virus gD protein using HEK-293T cells.

**Results:**

We describe in this study that individual gD proteins produced by a mammalian cell expression system are well immunogenic and stimulate high levels of PRV-specific and neutralizing antibodies in mice and piglets. All mice and piglets survived lethal doses of PRV, significantly reducing the amount of PRV virus in piglets’ lymph nodes, lungs, spleen, and other tissues. It also significantly reduced the time cycle and amount of viral excretion from piglets to the environment through the nasal and anal cavities.

**Discussion:**

The results suggest that PRV gD protein is expected to be a potential candidate for the preparation of genetically engineered PR vaccines for the prevention of PRV infection and the control of PR epidemics.

## 1. Introduction

Pseudorabies (PR) is caused by pseudorabies virus (PRV), which can infect both domestic animals (such as pigs, cattle, sheep, etc.) and wildlife (such as lynx, foxes, wild boars, etc.), Recent studies suggest that humans may also be potential hosts for this pathogen (Kong et al., 2013; Masot et al., 2017; Minamiguchi et al., 2019; Cheng et al., 2020). PRV, also known as suid herpesvirus (SuHV-1) or Aujeszky’s disease virus (ADV), belongs to the herpesvirus subfamily of the herpesvirus family (Nauwynck et al., 2007; Ai et al., 2018; Liu et al., 2021). Pigs are the only known natural hosts of PRV, which has a diverse host spectrum. PRV can be transmitted through the respiratory tract, digestive tract, and seminal placenta, and clinical manifestations of infection in pigs are diarrhea, vomiting, and neurological disorders (Mettenleiter, 1996; Marcaccini et al., 2008). It will disrupt the reproduction of maternal pigs, slow the growth of fertile pigs, and increase the incidence of piglets, making PR removal more difficult and causing huge losses to the world pig industry (Sun et al., 2016; Verpoest et al., 2017; Jiang et al., 2020).

PRV is a double-stranded DNA virus with a viral genome of about 145 kb that encodes 70–100 proteins, the majority of which are capsid proteins, envelope proteins, epidermal proteins, and enzymes (Mettenleiter, 2000). Eleven glycoproteins (gB, gC, gD, gE, gG, gH, gI, gK, gL, gM, and gN) and four transmembrane proteins (UL20, UL43, US9, and UL24) were identified on the virion envelope (Granzow et al., 2001). In the course of infection, gC, gB, gD, gH, and gL participate in the invasion of the virus and are the main antigens, which stimulate the host’s innate immune response (Kramer et al., 2011; Ye et al., 2015). Additionally, gD identifies and binds to molecules that resemble immunoglobulin (Ig), including connexin-1, connexin-2, and acetyl heparan sulfate (HS), which have a similar affinity to nectin-1 in humans and pigs (Li et al., 2017). Notably, PRV gD is the typical viral ligand for α herpesvirus entry into the host cell, Viral invasion of cells depends on the binding of gD to cell surface receptors (Krummenacher et al., 2005; Petrina et al., 2021). Moreover, gD is the main glycoprotein of PRV, which can stimulate the body to produce neutralizing antibodies against PRV infection (Fusco et al., 2005; He et al., 2019). PRV gB is essential for virus entry and transmission across cells, and gD is required for receptor engagement, stabilization of viral particle-cell interactions, and further activation of gB to become required for fusion competence (Hochrein et al., 2004; Oku et al., 2021). Growing evidence suggests that gD is a crucial protein for activating both humoral and cellular immune responses, making it a promising target for the development of new vaccines (Freuling et al., 2017; Aschner and Herold, 2021).

Due to the continuous evolution of PRV strains, currently available attenuated and inactivated vaccines do not provide adequate protection for pigs. Therefore, researchers are exploring subunit vaccines as a potential avenue for novel vaccines. Evidence from previous research indicates that subunit vaccines producing PRV gC and gD proteins in *Bacillus subtilis* may successfully induce a mucosal immune response, and the vaccine initiates the immune system more effectively than conventional vaccines in the presence of maternal immunity (Wang et al., 2019). PRV-gD mRNA triggered specific neutralizing antibodies, significantly higher cytokine IFN-γ/IL-2 levels than controls, and a considerable rise in the percentage of CD4^+^/CD8^+^ cells in peripheral lymphocytes, therefore protecting mice against PRV (Jiang et al., 2020). Expression of gB, gC, and gD through the baculovirus system can provide better protection for piglets. At 7 days post-immunization, piglets in the gD and gB + gD groups produced the highest NAs. After challenge with the PRV-HNLH mutant strain, none of the piglets showed clinical signs, such as elevated body temperature, and viral load and pathological damage were significantly reduced. In addition, the duration of gD vaccine-induced NAs was maintained for 4 months after a single vaccination (Zhang et al., 2020). By constructing chimeric viruses, it was found that injecting gC or gD may create excellent immunological effects and protect piglets against PRV-HB1201 challenge (Ren et al., 2020). Cao Z used baculovirus and *Escherichia coli* expression systems to express gB, gD, and GM-CSF, respectively. The inoculated rabbits had normal body temperatures, less pathological tissue damage, and a significantly lower viral load in tissues (Cao et al., 2022).

As science and technology have advanced, more and more viral proteins with favorable immunogenicity have been thoroughly investigated by scientists. The recombinant porcine circovirus type 2 VP2 protein and swine fever virus E2 protein have been shown to provide effective protection for piglets. Compared with prokaryotic expression, mammalian cells can correctly process both self-expressed and exogenous proteins and are the expression system of choice for obtaining highly active proteins *in vitro*. To develop a more effective vaccine against PRV variants, we generated gD protein using the HEK-293T expression system, and the immunization effect of gD protein in mice and piglets was evaluated.

## 2. Materials and methods

### 2.1. Viruses, cells, and antibodies

HEK-293T cells were grown in Dulbecco’s Modified Eagle Medium (DMEM; Gibco) with 10% fetal bovine serum (FBS; Gibco). It was maintained at 37°C and 5% carbon dioxide (CO_2_) incubator. PRV-HY was isolated from a pig farm in Guangdong, China, where a PR outbreak occurred. We purchased Mouse monoclonal antibody from Shenzhen Kejie Industrial Development that was tailored to the PRV gE.

### 2.2. Construction of the expression plasmids

Porcine pseudorabies glycoprotein D (gD) gene (GenBank: QEY95774.1) with a hexa-His tag to the C terminus for further purification processes was cloned into the lentiviral expression plasmid pLV-CMV-eGFP by restriction enzyme, the resulting plasmid was designated as pLV-CMV-gD. HEK-293T cells with robust and stable expression of recombinant gD protein was established by infection with recombinant lentivirus vector pLV-CMV-gD as previously described (Chen et al., 2021). A total of 4 × 10^4^ HEK 293T cells /well were prepared in a 24-wells plate. On the following day, cells in each well were infected with packaged recombinant lentivirus pLV-CMV-gD at a MOI of 10 in DMEM medium containing 10% FBS with 6 ~ 8 μg/ml hexadimethrine bromide (Polybrene, Sigma, Germany) 0.24 h after infection, cell culture media was replaced with fresh DMEM with 10% FBS and for 3 ~ 5 days at 37°C and 5% CO_2_, the optimal cell clone was selective and named HEK 293T-gD. Recombinant gD protein in the supernatant of cell cultures was collected and purified with Ni NTA resin affinity chromatography (GE Healthcare, US). In a nutshell, using a GE AKTA Pure system, 100 ml of culture supernatants were filtered through a 0.22 μm filter and put onto a 5 ml Ni-NTA column (GE) that had been equilibrated in 20 mM Tris [pH 8.0]. Unbound proteins were cleaned off the column by washing it with washing buffer (50 mM NaH_2_PO_4_, 300 mM NaCl, 20 mM imidazole). The hexa-His-tagged recombinant protein was eluted using elution buffer containing 50 mM NaH_2_PO_4_, 300 mM NaCl, and 250 mM imidazole. SDS-PAGE was used to verify the pure protein, and the BCA Protein Assay Kit (Thermo, USA) was used to quantify it according to the manufacturer’s instructions. For animal immunization, the purified protein was diluted to 100 μg/ml in PBS and mixed with an equal amount of Montanide (TM) ISA 201 VG oil adjuvant (Seppic; 1 ml + 1 ml).

### 2.3. Immunization scheme

**Figure 1:**
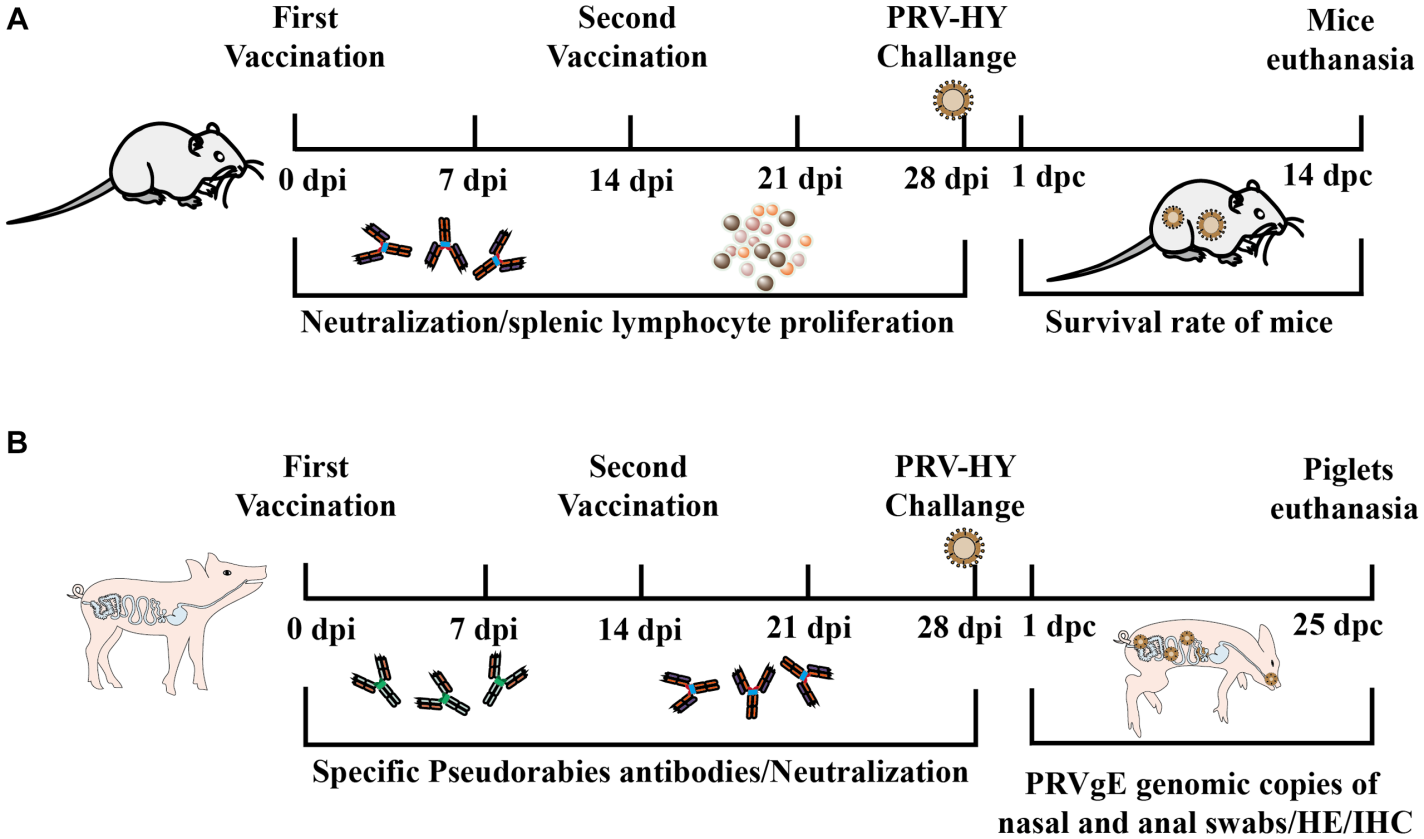
Experimental procedure for immune protection in mice and piglets. **(A)** Schematic diagram of antigen inoculation, challenge, and sample collection in mice. **(B)** Schematic diagram of piglet inoculation antigen, challenge, and experimental sample collection.

The purified gD protein was diluted to 50 μg/ml and then used in immunization tests in mice and piglets. Mice were immunized with subcutaneous multipoint injection on 0 and 14 days. Blood was collected from mice at 7, 14, 21, and 28 days after the first immunization, and the levels of PRV-specific and neutralizing antibodies were measured. At the end of the experiment, the surviving mice were euthanized (Figure 1A). Piglets were immunized by intramuscular injection at 0 and 14 days. Blood was collected from piglets at 7, 14, 21, and 28 days after the first immunization, and serum levels of PRV gD antibodies and neutralizing antibodies were measured and challenged against PRV-HY at 14 and 28 days. After the challenge, the rectal temperatures of piglets were measured daily, and nasal and anal swabs of piglets were collected every other day to determine the PRV gE gene copy number. At the end of the experiment, surviving piglets were euthanized and tissue samples were collected for HE and IHC experiments (Figure 1B).

### 2.4. Animal experiments

Four-week-old mice (*n* = 40) were divided into three groups. After acclimation, immunized mice with equal amounts of gD protein, commercial porcine inactivated vaccine (PCIV; Keqian Biological Company, Wuhan, China), DMEM, and PBS, the groups and doses are shown in Table 1. The gD group was immunized with 10 μg gD protein and subsequently immunized for the second time at 14 days, in addition to the PBS group, all mice were challenged with 10^3^ TCID_50_ PRV-HY at 28 days post-immunization. Every other week, the tails of the mice are amputated for blood collection and serum isolation. Mice were infected with PRV-HY at 28 days after the first inoculation and monitored for 14 days to determine survival. Each dead mouse was euthanized by intraperitoneal injection of pentobarbital sodium (200 μg/g).

**Table 1:** Immunization strategy in mice.

Group	Formulation	Immunization time points (DAI)	Immunization pathway	Vaccine dose
1	gD	0.14	Subcutaneous immunization	200 μl
2	commercial vaccine	0.14	Subcutaneous immunization	200 μl
3	DMEM	0.14	Subcutaneous immunization	200 μl
4	(PBS) negative control	0.14	Subcutaneous immunization	200 μl

Twenty healthy piglets at 4 weeks of age were randomly assigned to four groups: gD group, commercial vaccine group, DMEM group, and PBS group; the groups and doses are shown in Table 2. Antigens and antibodies of PRRSV, CSFV, PRV, and PCV2 in the serum of all piglets were negative. Each group was given 2 ml of gD protein, or commercial vaccine, DMEM culture media, and PBS through the muscle. Following the initial vaccination, piglets’ eating habits and rectal temperature were regularly monitored. Sera was isolated from piglets’ anterior vena cava blood to assess PRV-specific and neutralizing antibody levels. In addition to the PBS group, all piglets were infected with 10^7^ TCID_50_ PRV-HY through the nasal cavity at 28 days post-immunization. Piglet rectal temperatures should be taken once daily; clinical signs such as piglet feeding should be observed; and nasal and fecal swabs should be taken at 1, 3, 5, 7, 9,11,18, and 25 days post-challenge. Use qPCR to determine viral load. At 25 days post-challenge, the surviving piglets were euthanized by intravenous injection of pentobarbital sodium (100 mg/kg), Illness samples were collected, either fixed in 4% formalin zinc fixative (Sigma Aldrich) for histopathology and immunohistochemistry detection or frozen at −80°C for eventual viral gene copies assessment.

**Table 2:** Immunization strategy in piglets.

Group	Formulation	Immunization time points (DAI)	Immunization pathway	Vaccine dose
1	gD	0.14	Intramuscular injection	2 ml
2	Commercial vaccine	0.14	Intramuscular injection	2 ml
3	DMEM	0.14	Intramuscular injection	2 ml
4	(PBS) Negative control	0.14	Intramuscular injection	2 ml

### 2.5. Neutralizing antibody test

Sera was tested for neutralizing PRV-HY antibodies using Vero cells. Serum samples from mice and piglets were heat-inactivated at 60°C for 30 min and then diluted with DMEM. A similar amount of PRV-HY (200 TCID_50_) was mixed thoroughly with 50 μl of the diluted, inactivated serum. The mixture was then added to a 96-well plate with monolayer Vero cells. Positive serum and blank cells were set up as controls and cultured in DMEM containing 2% fetal bovine serum for observation. The plates were incubated for 5 days at 37°C in a 5% carbon dioxide environment to check for cytopathic effect (CPE). Neutralizing antibodies were calculated using the Reed-Muench method.

### 2.6. Lymphocyte isolation and stimulation

Lymphocytes were extracted from the spleen of mice 14 and 28 days after the first immunization. Check for cell division using the CCK-8 assay. Separate mouse spleen lymphocytes, then add 10^5^ cells per well to a 96-well plate with the ConA. After 48 h of incubation at 37°C, 10 μl of CCK-8 was added to each well, followed by an additional 2 h of incubation at 37°C. At a wavelength of 450 nm, absorption was determined for each well. The stimulation index (SI) indicates the proliferation level of mouse spleen cells.

### 2.7. Quantification of viral loads

Using a viral DNA/RNA kit, extract viral DNA from the sample and set up the reaction apparatus according to ChamQTM SYBR qPCR Master Mix instructions. Using qPCR, determine copies of the PRV gE gene in the liver, lungs, and other piglet organs. The primer sequences used for amplification were: upstream: 5′-GTCTGTGAAGCGGTTCGTGAT-3′ and downstream: 5′-ACAAGTCAAGGCGCATCTAC-3′. A standard curve was generated using a series of 7 dilutions containing the gE gene at copy numbers of 10^2^ to 10^8^ copies/μl as a template. The qPCR settings were 50°C for 2 min, 95°C for 2 min, 95°C for 15 s, 60°C for 15 s, and 72°C for 45 s for 40 cycles.

### 2.8. Histopathology and immunohistochemistry

After euthanasia, all animals were assessed for gross tissue damage to the lungs, lymph nodes, kidneys, tonsils, and brain during autopsy examinations. Three piglet organ samples were treated with 4% formaldehyde, prepared in paraffin, and subsequently frozen. To stain the slices with HE (Solarbio, China), they were dewaxed and washed. Then, for 20 min at 37°C, soak in 3% H_2_O_2_, followed by an hour of blocking in 37% horse serum. Slices were washed three times in PBS, then incubated with PRV gE monoclonal antibody (1:200) at 37°C for 30 min, and then at 4°C overnight. The slices are washed in PBS to get rid of any lingering antibodies, then incubated with HRP-labeled goat anti-mouse IgG (1,500) for an hour at 37°C. To detect antibody binding, use the HRPDAB chromogenic reagent kit purchased from Tiangen Biotechnology in Beijing, China. Use the Leica DMI3000B microscope (manufactured by Leica in Wetzlar, Germany) to take photographs.

### 2.9. Statistical analysis

One-way analysis of variance (ANOVA) was performed on the data between groups by using GraphPad Prism 9 (San Diego, CA, USA). Significance is presented as ^*^p < 0.05, ^**^p < 0.01, ^***^p < 0.001.

## 3. Results

### 3.1. Identification of gD protein expression in HEK-293T cells

**Figure 2:**
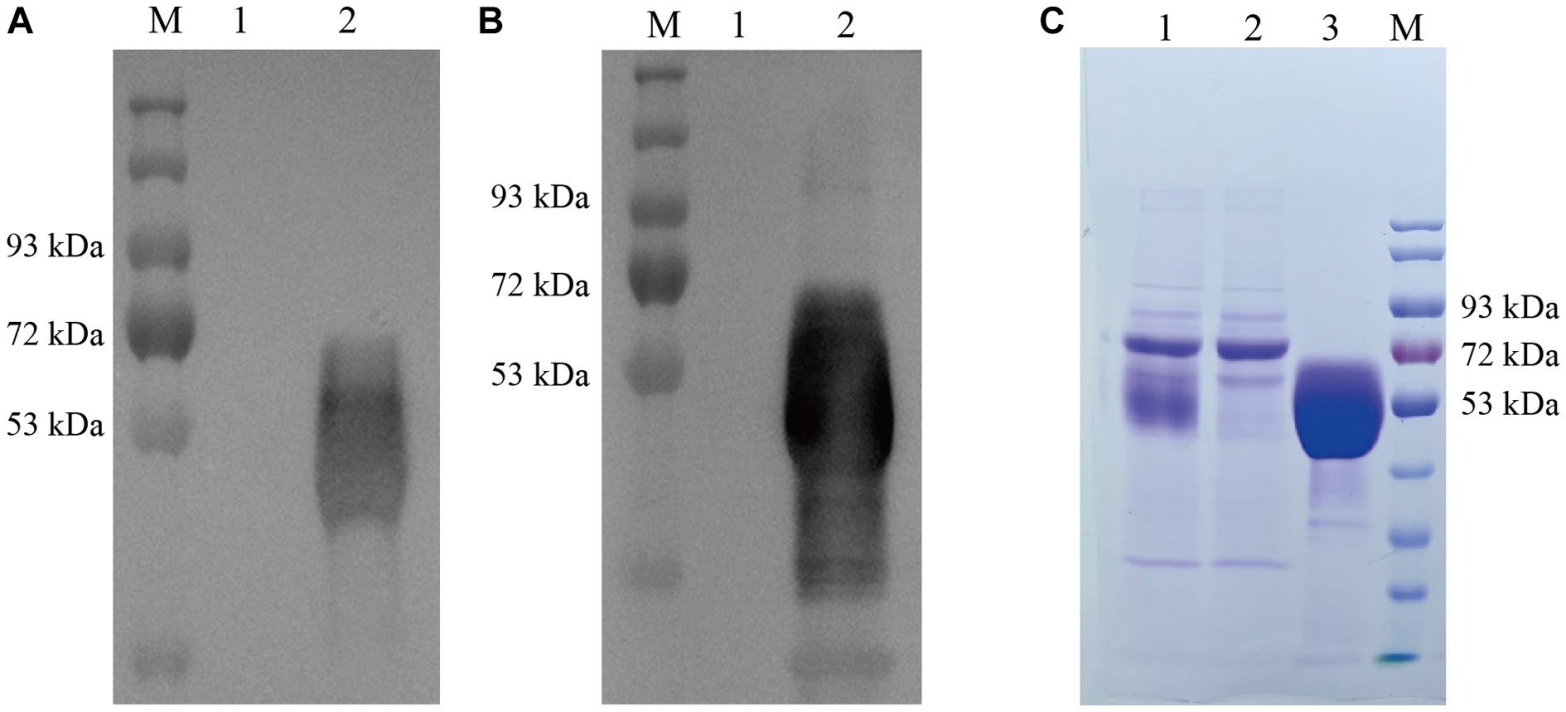
Identification and quantification of gD protein expression. Western blot detection of gD protein expression in HEK 293T-gD cell supernatant using anti his labeled antibody **(A)** and PRV high immune serum **(B)**. M: marker, lane 1: pLV-CMV-eGFP control, lane 2: pLV-CMV-gD. **(C)** SDS-PAGE was used to analyze the purified gD protein. Lane 1: crude culture supernatant; Lane 2: filtrate; Lane 3: eluate (500 mM imidazole); M: protein marker.

We obtained recombinant lentivirus using a lentiviral packaging system based on four plasmids (pPACKH1-GAG, pVSV-G, pPACKH1-REV, and pLV-CMV-eGFP). Secretory expression was achieved in mammalian HEK-293T cells by lentiviral mediation, and the recombinant gD protein was obtained by collecting cell cultures. The expression of gD protein in transfected cell supernatant was detected by Western blot using both anti-his antibody (Figure 2A) and anti-PRV hyperimmune serum (Figure 2B). The results showed that gD protein with the expected molecular weight (50 kDa) was efficiently expressed in HEK 293T-gD cells. Approximately 100 ml culture supernatants collected from HEK 293T-gD cells were purified by the BioLogic LP protein purification system using nickel affinity columns. The purified proteins were analyzed by experimental SDS-PAGE (Figure 2C), and the purified recombinant gD concentration was detected by the BCA Protein Assay Kit. The results showed that after the cell culture supernatant was purified by Ni NTA resin Affinity chromatography, the purity of gD was significantly improved, and the amount of gD protein reached 1.14 mg/ml.

### 3.2. Immunoprotective effect of PRV-gD in mice

**Figure 3:**
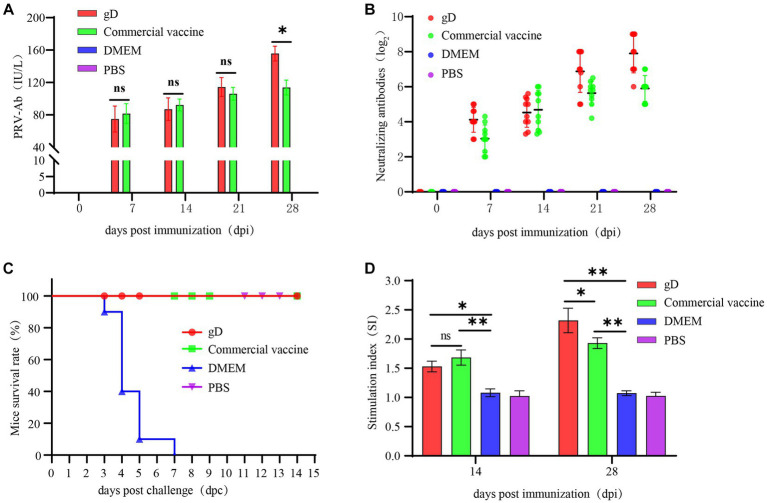
Protection against challenge with virulent PRV-HY strain in mice. Mice were inoculated with gD protein, commercial vaccine, DMEM, and PBS. All mice were immunized twice at 1 and 14 days and then challenged at 28 days with PRV-HY (10^3^ TCID_50_). Blood samples were collected weekly, and PRV-Ab antibodies **(A)** to gD and neutralizing antibodies **(B)** in mice sera were determined. **(C)** The survival rate of mice. **(D)** Lymphocyte proliferation level in mice. The data are representative of three independent experiments. Data were analyzed by One-Way ANOVA using GraphPad Prism 9. Significance is presented as ^*^p < 0.05, ^**^p < 0.01, ^***^p < 0.001.

To determine whether gD protein paired with ISA 201VG adjuvant protects mice from prevalent strains. At 7, 14, 21, and 28 days after mice were immunized with the vaccine, we measured PRV-specific antibody levels in mouse serum using the PRV-Ab antibody quantification kit and found that both the gD protein group and the commercial vaccine group reached higher levels after the initial immunization. At 28 days after immunization, PRV-Ab was significantly higher in the gD protein group than in the commercial vaccine group (Figure 3A). At 7 days after the first immunization, the neutralizing antibody reached a higher level; at 28 days after the booster immunization, the serum NAs potency of mice in the gD protein group reached 28, which was significantly higher than that of the commercial vaccine group (Figure 3B). The results indicated that the gD protein group could induce a higher level of humoral immune response within a shorter time after immunization. Mice were stimulated with ConA 14 and 28 days after immunization with the vaccine, and then their proliferation levels were detected by CCK-8. It was found that the stimulation index was significantly higher in both the gD protein group and the commercial vaccine group, and the stimulation index of the gD protein group was significantly higher than that of the commercial vaccine group (Figure 3D). The results indicated that the gD protein group was able to induce a higher level of cellular immune response within a shorter time after immunization. Mice were infected by intraperitoneal injection using a dose of 10^3^ TCID_50_ of PRV-HY at 28 days after vaccination. After the challenge with PRV-HY, typical clinical signs such as pruritus, neurological signs, and death were observed in the DMEM group of mice at 2–6 days post-challenge; on day 6 post-challenge, all mice in the DMEM group died (100%), while none of the other groups showed typical clinical signs and death (Figure 3C). Overall, gD protein stimulated mice to produce higher levels of PRV-Ab, NAs, and lymphocytes, and could completely protect mice from lethal doses of PRV infection.

### 3.3. Immunoprotective effect of PRV-gD in piglets

**Figure 4:**
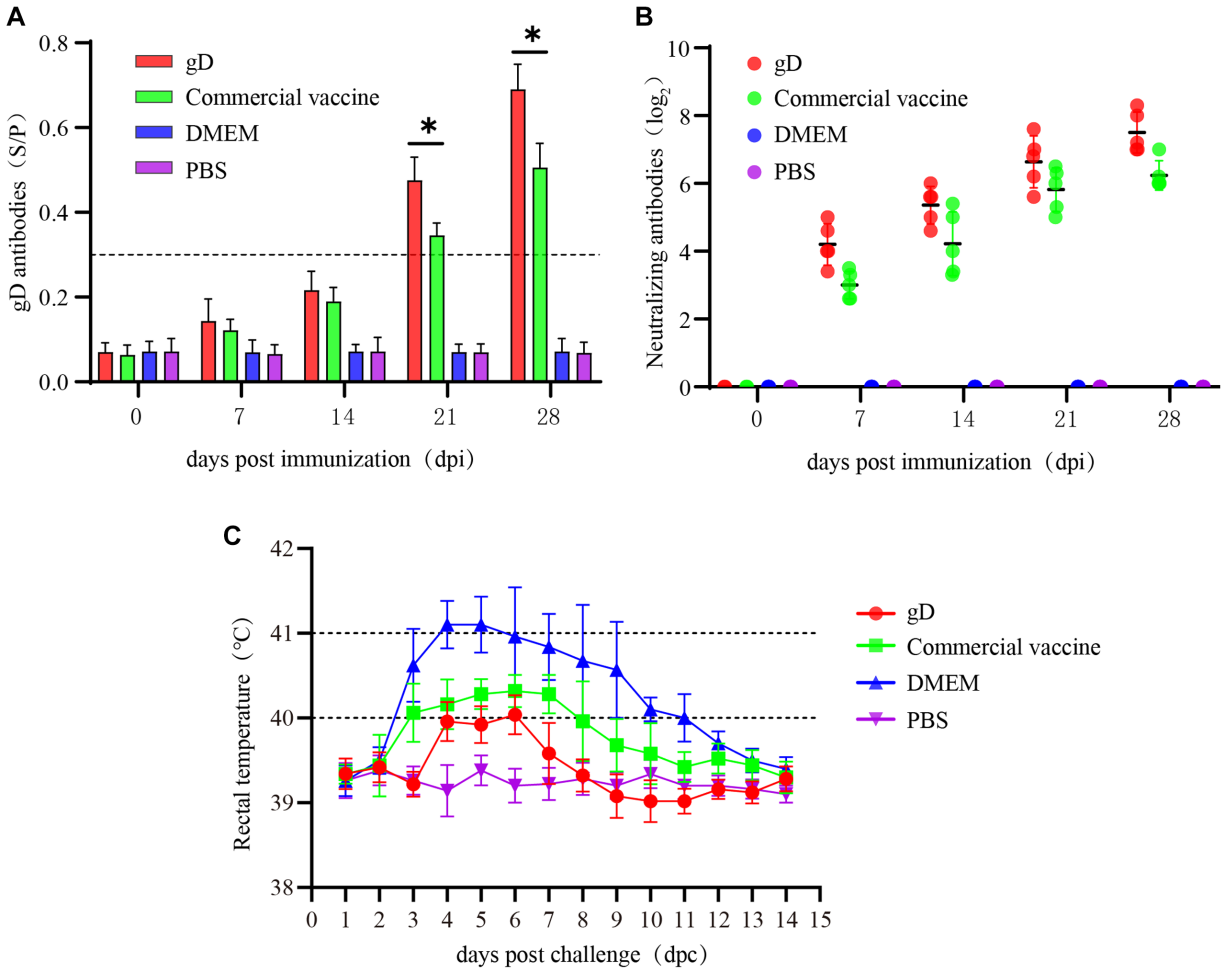
Blood samples were collected weekly after immunization in Piglets. Serum levels of gD and gB antibodies were measured at different time points. **(A)** gD antibodies were determined for piglet serum. **(B)** NAs titers in piglets at different times after immunization with the vaccine. **(C)** Rectal temperature of each group of immunized piglets after lethal dose challenge of PRV-HY. Data were analyzed by one-way ANOVA using GraphPad Prism 9. Significance is expressed as ^*^p < 0.05, ^**^p < 0.01, ^***^p < 0.001.

**Figure 5:**
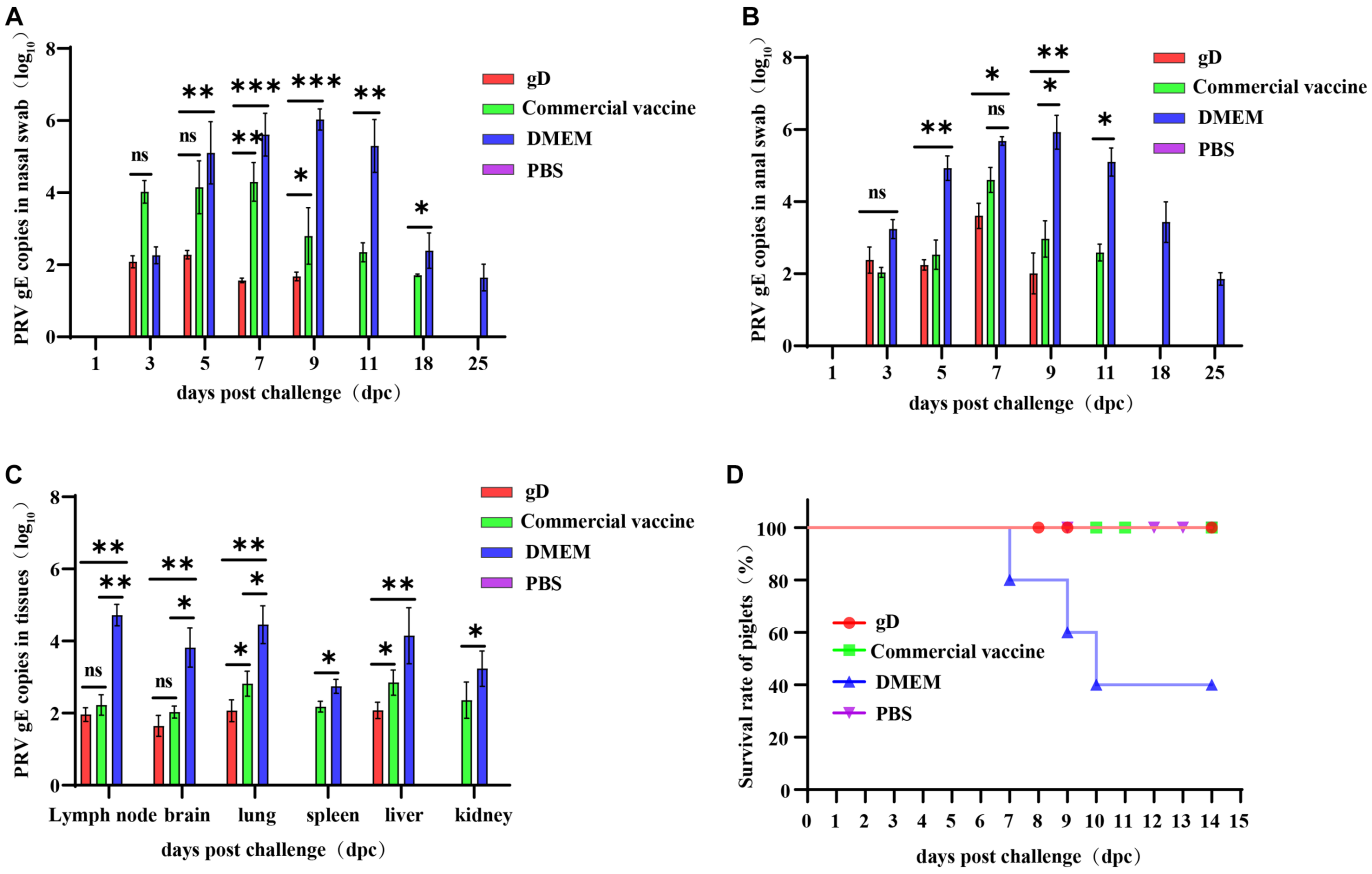
Protection against challenge with virulent PRV-HY strain in piglets. Absolute fluorescence quantification of PRV gE gene on oral swabs, anal swabs, and tissues from three groups of piglets. Nasal swabs **(A)** and anal swabs **(B)** were collected from piglets after the challenge, and qPCR amplification was performed to detect PRV gE genomic copies (Log_10_ copies/g). **(C)** Tissues of piglets from each group were collected and qPCR was amplified to detect PRV gE gene copies (Log_10_ copies/g). **(D)** Survival of immunized piglets in each group after challenge with a lethal dose of PRV-HY. Data were analyzed by one-way ANOVA using GraphPad Prism 9. Asterisks indicate significant differences (^*^p < 0.05, ^**^p < 0.01, ^***^p < 0.001).

To further evaluate the protective effect of gD protein on piglets, 100 μg of gD protein was injected intramuscularly. At 7, 14, 21, and 28 days after piglets were vaccinated, we measured PRV gD antibodies in piglets’ sera using an indirect ELISA quantification kit, and the results were expressed as S/P values at 40-fold serum dilution. Twenty one days after immunization, both the gD protein group and the commercial vaccine group had reached high levels, and the gD protein group had significantly higher antibody levels than the commercial vaccine group (Figure 4A). Seven days after the initial immunization, both the gD protein group and the commercial vaccine group reached higher levels of neutralizing antibodies; 28 days after immunization, the potency of NAs titer in the serum of piglets in the gD protein group reached 2^7^, which was significantly higher than that of the commercial vaccine group (Figure 4B). The results showed that the gD protein group was able to induce a higher level of humoral immune response within a shorter time after immunization. Piglets were infected with the PRV-HY through the nasal cavity using a dose of 10^7^ TCID_50_ 28 days after vaccination. After the challenge, rectal temperature, clinical symptoms, and piglet mortality were measured daily. The results showed that the rectal temperature of piglets in the DMEM group continued to increase for 4 days, and the rectal temperature of piglets in the DMEM group exceeded 40.5°C. The rectal temperature of piglets in the gD protein, commercial vaccine, and PBS groups did not increase significantly (Figure 4C). We also observed that piglets in the DMEM group showed typical clinical signs such as severe respiratory problems, decreased appetite, convulsions, diarrhea, and recumbency, while the other groups showed no significant clinical signs. In addition, three piglets in the DMEM group died at 7, 9, and 10 days after the PRV-HY challenge (Figure 5D). In contrast, piglets in the gD protein group, the commercial vaccine group, and the PBS group survived without any significant CNS signs. Overall, the gD protein stimulates piglets to produce higher levels of PRV-gD antibodies and NAs, which could completely protect piglets against lethal doses of PRV-HY challenge.

### 3.4. Virus loads in nasal swabs, anal swabs, and tissues of the piglets

At 28 days after immunization, piglets were challenged with high doses of potent PRV-HY via the nasal route, and nasal swabs, anal swabs, and piglet tissues were collected to detect the PRV gE gene copy number by fluorescent quantitative nucleic acid amplification. The results showed that nasal and anal swabs from all groups of piglets were negative for the PRV gE gene at 1 day post-challenge. At 3–9 days after the challenge, the PRV gE gene was detected in piglet samples from all groups except the PBS negative control group, and the PRV gE gene copy number in the gD protein and commercial vaccine groups was significantly lower than in the DMEM group. On days 7 and 9, the nasal swab viral load was significantly lower in the gD protein group than in the commercial vaccine group (Figure 5A). In addition, the PRV gE gene could not be detected in nasal and anal swabs of the gD protein group on day 9 after the challenge. However, the PRV gE gene could still be detected in the nasal swabs of the commercial vaccine group on day 18 after the challenge, and the PRV gE gene could also be detected in anal swabs of the commercial vaccine group littermates on days 11 after the challenge (Figure 5B). At the end of the experiment, all piglets were euthanized and then pathologically dissected and tissue samples were collected to copies of the PRV gE gene in lymph nodes, brain, lung, spleen, liver, and kidney tissues. The results showed that the PRV gE gene was detected in piglet tissues from the commercial vaccine and DMEM groups, but only a small amount of the PRV gE gene was detected in the lymph nodes, brain, and lung tissues of piglets in the gD protein group, and the spleen and kidney were negative. In addition, the PRV gE gene copy number in all piglet tissues from the gD protein and commercial vaccine groups was significantly lower than in the DMEM group (Figure 5C). In summary, after the challenge, piglets in the gD protein group had lower viral shedding loads and shorter viral shedding times through the nasal and anal, which may be related to the fact that gD protein can stimulate the host to produce high levels of neutralizing antibodies.

### 3.5. Pathological examination of piglet tissues

**Figure 6:**
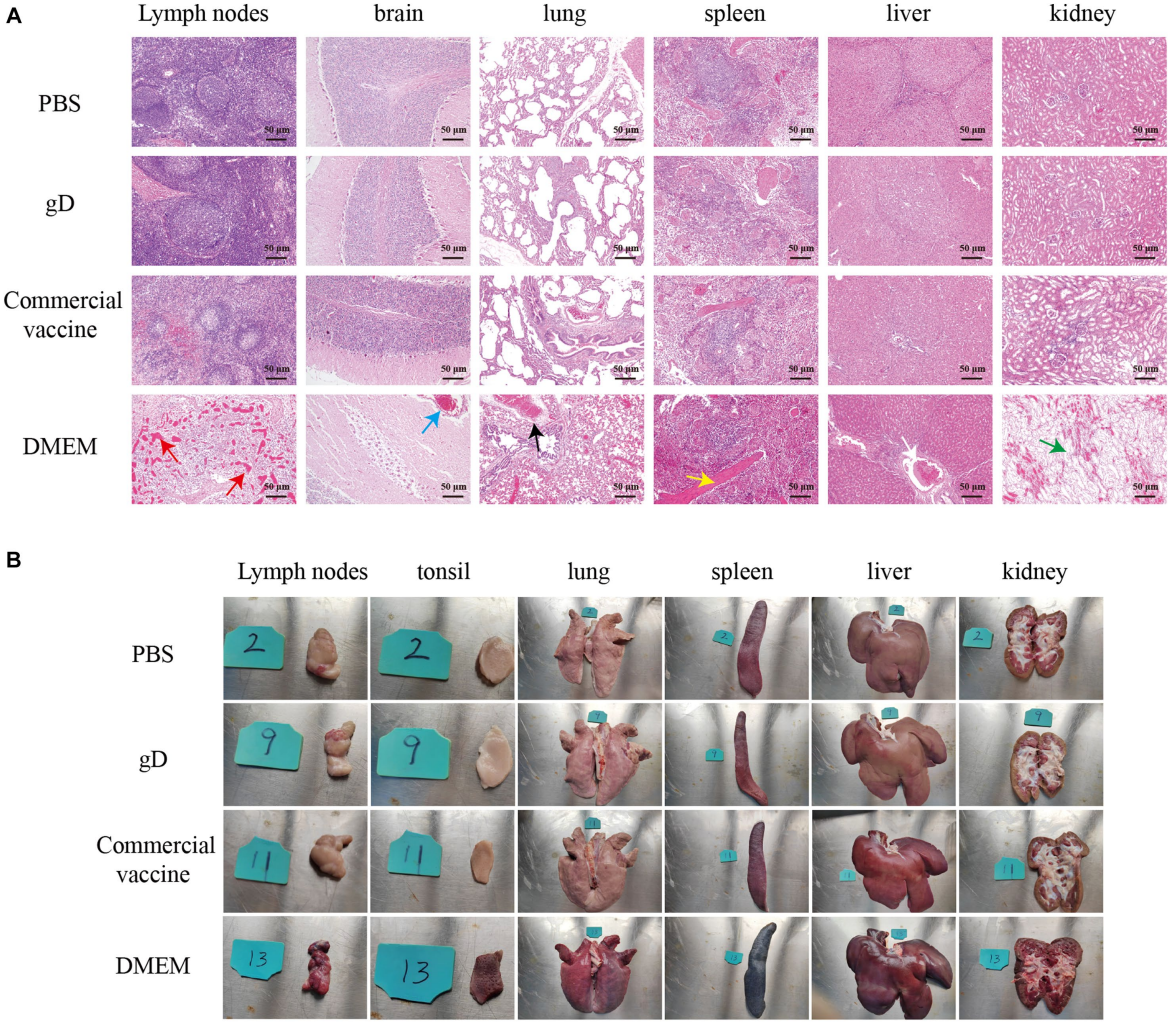
Overall changes in organ damage after challenging immunized pigs with PRV-HY. **(A)** Different piglet tissues (lymph nodes, tonsils, lungs, spleen, liver, and kidneys) were collected and euthanized on the 25th day after the challenge and dissected for pathological examination, here showing representative lesions in different organs. **(B)** Histopathological lesions rendering pigs immune after challenge with PRV-HY strain. The various tissues shown were fixed, segmented, and stained with hematoxylin and eosin (HE).

Twenty-five days after the challenge, the surviving piglets were euthanized by intravenous sodium pentobarbital (100 mg/kg), followed by necropsy and histopathological examination. Lymph nodes, tonsils, lungs, spleen, liver, and kidneys were essentially normal in PBS, gD protein, and commercial vaccine groups. Lungs of piglets in the DMEM group exhibited severe pulmonary bruising, pulmonary edema, and solid lung lesions; lymph nodes were bruised and enlarged; tonsils were severely present and edematous; spleen was hemorrhagic infarcted; liver had striated gray-white necrotic foci on the surface; and renal medulla and renal cortex were severely hemorrhagic (Figure 6A). Sections were stained with hematoxylin–eosin (HE) to observe histopathological changes. The results showed that compared with the PBS group, the gD protein group, and the commercial vaccine group, the piglets in the DMEM group had reduced lymphocytes in the submandibular lymph nodes and hemorrhage in the medulla; inflammatory cell infiltration and bruising in the brain capillaries; extensive alveolar epithelial cell hyperplasia with massive inflammatory cell infiltration in the lungs, significant widening of the alveolar diaphragm, and a small number of inflammatory cells visible in the lumen of the fine bronchi; necrotic tissue in the spleen, focal lymphocytes in the red medulla necrosis and excessive congestion in the spleen; cell proliferation in the liver was quite obvious; some glomeruli had inflammatory cell infiltration, capillary hyperplasia, and massive neutrophil infiltration, and some renal cystic lumens disappeared. The results showed that the gD protein prepared in this study significantly attenuated the lesions in lymph nodes, tonsils, lungs, spleen, liver, and kidneys caused by PRV-HY infection (Figure 6B).

### 3.6. Immunohistochemistry of piglet tissues

**Figure 7:**
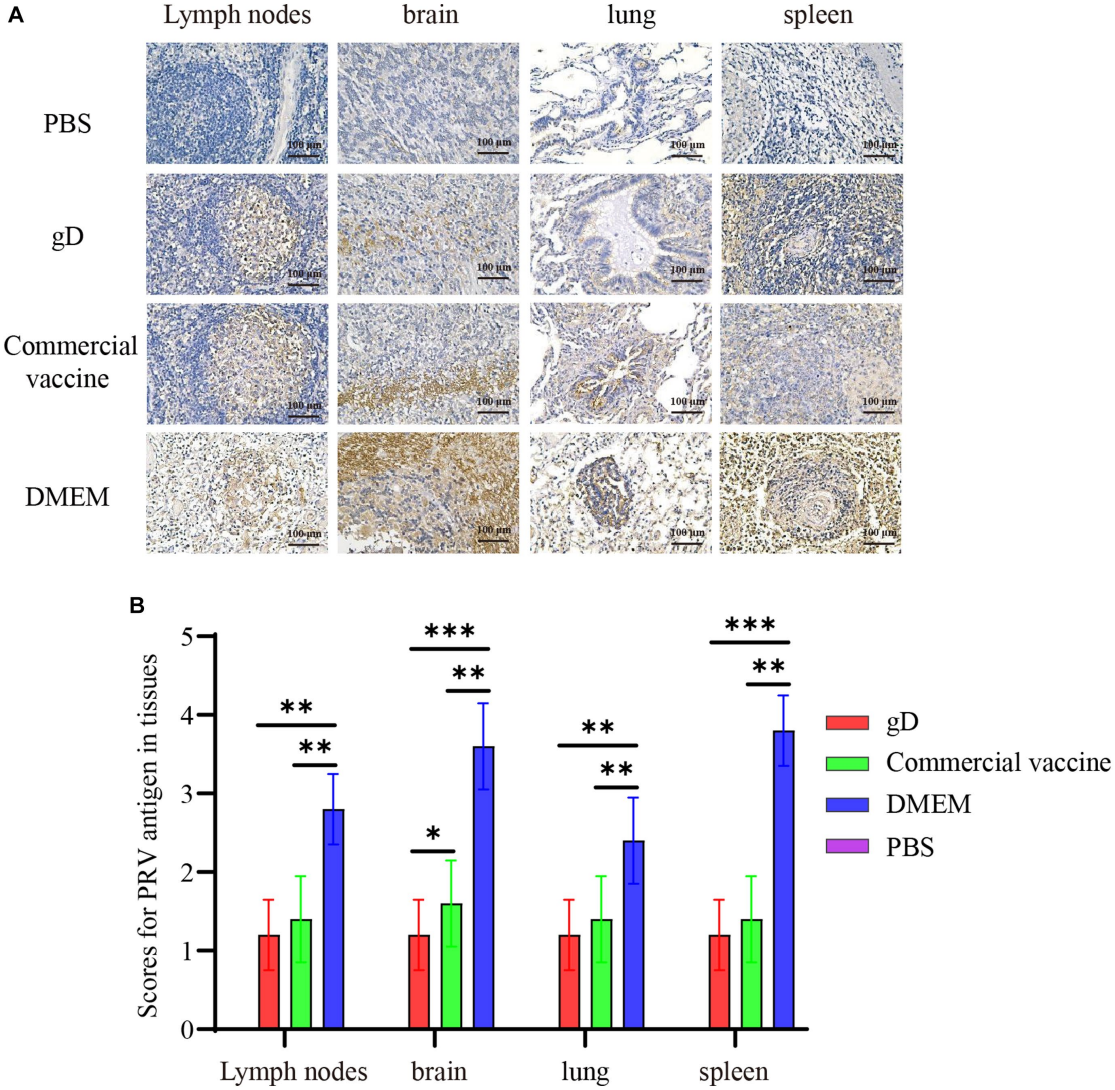
Immunohistochemical examination and scoring of piglet tissues. **(A)** Piglet lymph nodes, brain, lung, and spleen tissues were fixed, divided, and stained with PRV monoclonal antibody (1:400). Yellowish-brown color indicates a PRV-positive signal. **(B)** The percentage of positive cells was scored by ImageJ calculation. Asterisk indicates a significant difference: ^*^p < 0.05, ^**^p < 0.01, ^***^p < 0.001.

Immunohistochemistry (IHC) experiments on piglet tissues using PRV gE monoclonal antibody showed that a large number of PRV-infected tan cells were present in lymph nodes, brain, lung, and spleen tissues in the DMEM group, while the gD protein and commercial vaccine groups had a smaller number of PRV-infected cells and lighter staining (Figure 7A). In addition, we analyzed the IHC results using ImageJ to calculate the ratio of positive cells for each tissue and score them. The results showed that the gD protein group and the commercial vaccine group scored significantly lower than the DMEM group (Figure 7B). These findings suggest that the gD protein prepared in this study may reduce replication in PRV lymph nodes, brain, lung, and spleen tissues, thus protecting piglets from lethal doses of PRV-HY infection.

## 4. Discussion

PR is an acute infectious disease caused by PRV infection. Under the current situation in China, PRV-infected pigs occur from time to time, which has seriously jeopardized the development of the Chinese pig industry and caused significant economic losses to the Chinese pig industry (Freuling et al., 2017; Tan et al., 2021). Since late 2011, genetic recombination has occurred between vaccine and wild strains of porcine pseudorabies virus, resulting in a significant increase in virulence of newly emerged recombinant PRV strains, and new porcine pseudorabies virus strains can evade the protection of traditional inactivated and live vaccines, resulting in increased morbidity and mortality in pigs, posing new challenges for porcine pseudorabies disease prevention, control, and decontamination in China (Hu D. et al., 2015; Ye et al., 2016). Therefore, the development of safe and effective novel genetically engineered vaccines is essential for the prevention, control, and prevalence of porcine pseudorabies, and subunit vaccines have become a potential option due to their stability and safety.

The selection of proteins with good immunogenicity is essential for the development of genetically engineered vaccines. Numerous studies have shown that porcine pseudorabies gD protein facilitates viral adhesion to the host cell surface. gD is a key recognition receptor involved in pathogen binding that stimulates the host to produce high titers of neutralizing antibodies and exhibits good immunogenicity, and this molecular recognition pattern of gD protein is essential for PRV infection (Takahashi et al., 1993; Fan et al., 2014; Zhang et al., 2021). In addition, gD proteins can recognize and attach to immunoglobulin-like cell adhesion molecules, such as connexin-1, connexin-2, and acetyl heparan sulfate. In humans and pigs, Nectin-1 is the only receptor reported for PRV cell entry. gD may have a similar affinity to connexin-1, and gD-specific mAbs (10B6) exhibit potent inhibition of PRV cell attachment and prevent virus transmission between cells (Hammond et al., 2001; Krummenacher et al., 2005). One study replaced the gD gene of the PRV Bartha-K61 strain with the gD gene of the HB1201 strain, and the recombinant Bartha-K61 strain gave pigs strong humoral and cellular immunity and protected them from the lethal challenge of HB1201, suggesting that gD may induce cellular immunity in cross-protection (Ren et al., 2020). In addition, immunization of piglets with recombinant adenovirus-expressing porcine pseudorabies virus gD protein stimulated the host to produce high levels of neutralizing antibodies, and no viremia was detected in piglets after the PRV challenge (Brisse et al., 2020). These studies suggest that expression of porcine pseudorabies virus individual gD protein may well induce re-emergence to produce humoral and cellular immunity and reduce the PRV mutant strain against piglets. The gD protein, which is essential for porcine pseudorabies virus infection in host cells, can encode a protein closer to the natural protein structure using the full-length gD gene. Taking into account the conditions available in a fairly pre-laboratory setting, a human embryonic kidney cell line (HEK 293T-gD) that can stably express the gD protein was constructed by a lentiviral packaging system in this study. The results showed that the HEK 293T-gD cell line could secretly express porcine pseudorabies virus gD protein. In subsequent experimental animal studies, the gD protein was found to be sufficient to induce higher levels of humoral and cellular immunity in mice, protecting them from lethal doses of PRV-HY.

Compared to viral and whole virus vaccines, recombinant protein-based subunit vaccines are relatively stable and easy to produce using recombinant protein technology, making them an attractive vaccine platform (Manoj et al., 2004). In addition, the lack of an active viral component minimizes the disease risk of subunit vaccination, demonstrating an extraordinary safety profile. The HEK 293T mammalian cell expression system with high-efficiency promoter (CMV) control allows high expression of recombinant proteins, post-translational modification of proteins and correct protein folding complexation, and expression products with good immunogenicity and safety, which can compensate for the prokaryotic expression system expressed proteins with low purity, immunogenicity, and CHO expression system expressed proteins (Luckow et al., 1993). In this study, the nucleotide sequence of recombinant gD protein was optimized, His tag was introduced at the C-terminus of the target protein, and HEK 293T cells that could be secreted for protein expression were selected, and the final amount of purified PRV gD protein expression reached 1.14 mg/ml, and Western blot results showed that the resulting gD protein had good antigenicity. High levels of NAs could be detected in the serum of piglets immunized with gD protein 28 days after immunization, and some piglet serum-neutralizing antibodies have a titer of 2^8^.

To further evaluate the protective effect of gD protein on piglets against challenge, piglets were challenged by nasal drops 28 days after immunization. The results showed that all piglets in the gD protein group survived, indicating that gD protein induced immunity in piglets sufficient to resist lethal challenge by 10^7^ TCID_50_ PRV at a dose that was 10 to 100 times higher than the normal dose used. It is important to note that in contrast to previous studies, Hu found that piglets inoculated with the rSMXgI/gE1TK attenuated drug line (at a dose of 10^6^ TCID_50_) had a fever lasting 4 days and rectal temperatures at 40 and 42°C after challenge with the PRV variant SMX strain at 10^7^ TCID_50_ (Hu R.-M. et al., 2015). In this study, after the challenge, piglets in the gD protein group showed no typical clinical signs and all piglets did not develop fever, while some piglets in the commercial vaccine group epidemic had rectal temperatures above 40.5°C. The results of the study by Ren, J showed that two piglets in the Bartha-K61 group that died after the challenge exhibited severe hemorrhage in the lungs, lymph nodes, and kidneys, pulmonary coagulation, and cerebral edema. Piglets immunized with Bartha-gCHB1201 and Bartha-gDHB1201 showed moderate or mild hemorrhagic and coagulopathic lesions in the lungs and only mild lymph node enlargement (Ren et al., 2020), and these results were consistent with our results (Figure 6A). In addition, the number of viral gene copies in nasal swabs and anal swabs of piglets in the gD group significantly resisted the commercial vaccine group and had a shorter detoxification cycle to the environment. Compared to the commercial vaccine group, piglets in the gD group showed fewer pathological changes in lymph nodes, lungs, spleen, liver, and kidneys, and fewer positive cells in tissues were infected with PRV. We speculate that this may be due to the ability of the gD protein to rapidly induce higher levels of neutralizing antibodies in the host and that PRV-neutralizing antibodies can neutralize most of the virus when it enters the peripheral blood. On the other hand, the ISA 201 adjuvant effectively stimulates the host to produce strong mucosal immunity in concert with the gD protein, and these speculations need to be investigated in our subsequent studies. Based on this, the genetically engineered PR vaccine prepared from the HEK 293T expression system is a potential vaccine candidate for the prevention of PRV infection and the control of PR epidemics. Although the preliminary results of this study demonstrated that PRV gD protein could induce high titers of neutralizing antibodies in mice and piglets, there was no in-depth discussion on the organism’s level of cellular immunity, whether it could protect against different PRV strains, and the mechanism of the immune effect exerted by the gD protein, which is the direction of our future work.

## 5. Conclusion

In conclusion, we describe in this study that individual gD proteins produced by a mammalian cell expression system are well immunogenic and stimulate high levels of PRV-specific and neutralizing antibodies in mice and piglets. All mice and piglets survived lethal doses of PRV, significantly reducing the amount of PRV virus in piglets’ lymph nodes, lungs, spleen, and other tissues. It also significantly reduced the time cycle and amount of viral excretion from piglets to the environment through nasal and anal cavities. The results suggest that the PRV gD protein is expected to be a potential candidate for the preparation of genetically engineered PR vaccines for the prevention of PRV infection and the control of PR epidemics.

## Data availability statement

The original contributions presented in the study are included in the article/supplementary material, further inquiries can be directed to the corresponding authors.

## Ethics statement

The animal study was approved by Experimental Animal Ethics Committee of Institute of the Animal Health, Guangdong Academy of Agricultural Science. The study was conducted in accordance with the local legislation and institutional requirements.

## Author contributions

MZ: Data curation, Visualization, Writing – original draft. JC: Writing – review & editing, Data curation. SL: Writing – review & editing. RY: Methodology, Writing – review & editing. PZ: Validation, Writing – review & editing. ZR: Software, Writing – review & editing. XC: Writing – review & editing. GW: Funding acquisition, Writing – review & editing. HX: Writing – review & editing. RC: Writing – review & editing. YH: Writing – review & editing. NL: Writing – review & editing. HL: Writing – review & editing. Z-GY: Resources, Supervision, Writing – review & editing. XW: Funding acquisition, Writing – review & editing.
